# Quantifying the impact of the COVID‐19 pandemic on gastrointestinal cancer care delivery

**DOI:** 10.1002/cnr2.1427

**Published:** 2021-06-17

**Authors:** Nicholas R. Perkons, Casey Kim, Chris Boedec, Luke J. Keele, Charles Schneider, Ursina R. Teitelbaum, Edgar Ben‐Josef, Peter E. Gabriel, John P. Plastaras, Lawrence N. Shulman, Andrzej P. Wojcieszynski

**Affiliations:** ^1^ Perelman School of Medicine Philadelphia Pennsylvania USA; ^2^ Data Analytics Center Perelman School of Medicine Philadelphia Pennsylvania USA; ^3^ Department of Biostatistics, Epidemiology, and Informatics Perelman School of Medicine Philadelphia Pennsylvania USA; ^4^ Department of Medicine Perelman School of Medicine Philadelphia Pennsylvania USA; ^5^ Department of Radiation Oncology Perelman School of Medicine Philadelphia Pennsylvania USA

**Keywords:** colonoscopy, gastrointestinal neoplasms, oncology service, hospital

## Abstract

**Background and Aim:**

This study quantifies how changes in healthcare utilization and delivery during the first months of the COVID‐19 pandemic have altered the presentation, treatment, and management of patients with gastrointestinal (GI) malignancies within an academic health system.

**Methods and results:**

Patients diagnosed with a GI malignancy (ICD10: C15‐C26) who received medical care within the health system during the observation period (first 44 weeks of 2019 and 2020) were identified for a retrospective cohort study. Deidentified patient encounter parameters were collected for this observation period and separated into pre‐pandemic (weeks 1–10) and early pandemic (weeks 11–20) study periods. Difference‐in‐difference analyses adjusted for week‐specific and year‐specific effects quantified the impact of the COVID‐19 pandemic on care delivery between pre‐pandemic and early pandemic study periods in 2020. Across all GI malignancies, the COVID‐19 pandemic has been associated with a significant decline in the number of patients with new patient visits (NPVs) (*p* = 1.2 × 10^−4^), Radiology encounters (*p* = 1.9 × 10^−7^), Surgery encounters (*p* = 1.6 × 10^−3^), Radiation Oncology encounters (*p* = 4.1 × 10^−3^), and infusion visits (6.1 × 10^−5^). Subgroup analyses revealed cancer‐specific variations in changes to delivery. Patients with colorectal cancer (CRC) had the most significant decrease in NPVs (*p* = 7.1 × 10^−5^), which was significantly associated with a concomitant decrease in colonoscopies performed during the early pandemic period (r^2^ = 0.722, *p* = 2.1 × 10^−10^).

**Conclusions:**

The COVID‐19 pandemic has been associated with significant disruptions to care delivery. While these effects were appreciated broadly across GI malignancies, CRC, diagnosed and managed by periodic screening, has been affected most acutely.

## 1 INTRODUCTION

In responding to the COVID‐19 pandemic, health care systems have been forced to redefine established processes of care delivery in order to balance local surges in COVID‐19 patient volumes, infection risk, resource limitations, and the baseline needs of existing patient populations. This has particular relevance for cancer care as health systems balance benefits and risks of frequent clinical encounters to cancer patients, who are thought to be at increased risk of mortality and complications from COVID‐19.^1^, ^2^ Although cancer patients traditionally have frequent points of contact with the healthcare system for clinical visits, imaging, procedures, and infusion sessions, this care delivery model has been significantly disrupted by the COVID‐19 pandemic and implementation of infection prevention measures in hospitals. In the early pandemic, many national healthcare systems and professional societies limited or delayed screening procedures and elective surgeries.^3^ However, many clinical decisions for cancer patients have been decided on an individual basis, and few studies quantify the degree of change and impact of these decisions on diagnosis, treatment, and outcomes. Furthermore, additional time and follow‐up will be needed to fully understand exactly how COVID‐19‐related changes to care have affected long‐term patient outcomes. This study seeks to quantify how the first months of the COVID‐19 pandemic have affected the presentation, treatment, and management of patients with gastrointestinal (GI) malignancies in an academic medical center.

## 2 PATIENTS AND METHODS

Patients with GI malignancies were identified by the International Statistical Classification of Diseases and Related Health Problems, Tenth Revision (ICD‐10) principal diagnosis codes (ICD10: C15‐C26). The electronic medical record was queried for deidentified encounter data for patients with a documented GI malignancy, including the weekly number of patients with new patient visits (NPVs) and/or specialty encounters during a 44‐week observation period in 2019 and 2020. Specialty encounters were isolated by searching for either an encounter department (Radiology, Surgery, Radiation Oncology) or encounter type (infusion visit) of interest. The study period, which includes the first 20 weeks of 2020 (1/1–5/20), was split into two subdivisions, the pre‐pandemic period (weeks 1–10, 1/1–3/17) and the early pandemic period (weeks 11–20, 3/18–5/19). Difference‐in‐difference analyses adjusted for week‐specific and year‐specific effects were used to compare encounter details between the early pandemic and pre‐pandemic periods of 2020 with weeks 1–20 of 2019 as an additional control. Within this framework, the difference‐in‐difference analysis quantified the impact of the COVID pandemic on health encounters by building a linear model (with encounter data from 2019 and the pre‐pandemic period of 2020) to isolate the otherwise unexplained decline in the number of patients with encounters during the early pandemic period of 2020. When not otherwise specified, P‐values reflect the probability that an observed change in the frequency of patients with an encounter during the early pandemic period could be ascribed to chance after accounting for week‐specific and year‐specific effects in a linear model.

A subset of encounter features was analyzed for patients with a GI malignancy, as defined by ICD10 code (C15‐C26). Subgroups were defined as follows: anal cancer (C21.*), biliary cancer (C22.1, C23.*, C24.*), colorectal cancer (CRC, C18.*, C19.*, C20.*), esophageal cancer (C15.*), gastric cancer (C16.*), hepatocellular carcinoma (HCC, C22.0), non‐HCC liver cancer (22.2, 22.3, 22.4, 22.7, 22.8, 22.9), pancreatic cancer (C25.*), and small intestine or otherwise non‐specified GI cancers (henceforth grouped together as *other GI cancers*; C17.*, C26.*) [* is used to denote all sub‐indexes of a given index]. In addition, the number of colonoscopies performed on all patients within the health system was recorded during the observation period. All analyses were performed using RStudio 1.1.442.

The institutional review board at the “Blinded for peer review” reviewed this study and waived patient informed consent.

## 3 RESULTS

Twenty‐five thousand seven hundred sixty‐six patients with a diagnosed GI malignancy were identified for the 2020 study cohort and compared to 23 530 patients in the 2019 cohort. Patient demographics are shown in Table 1. There was no statistically significant difference between cohorts on the basis of sex (χ^2^ = 0.15, *p* = .93) nor race (χ^2^ = 2.82, *p* = .99).

**TABLE 1:** Cohort demographics for patients with gastrointestinal cancer

	No. (% of Patients)	
Characteristics	2020 Cohort^a^	2019 Cohort^a^
*Overall*	25 766	23 530
*Sex*		
M	14 673 (56.9)	13 360 (56.8)
F	11 092 (43.0)	10 169 (43.2)
Unknown	1 (0.0)	1 (0.0)
*Race/Ethnicity*		
White	18 108 (70.3)	16 566 (70.4)
Black	4208 (16.3)	3844 (16.3)
Asian	789 (3.1)	687 (2.9)
Hispanic Latino/White	367 (1.4)	321 (1.4)
Hispanic Latino/Black	144 (0.6)	122 (0.5)
East Indian	94 (0.4)	79 (0.3)
Pacific Islander	27 (0.1)	25 (0.1)
American Indian	27 (0.1)	24 (0.1)
Other	1079 (4.2)	1016 (4.3)
Unknown	1019 (4.0)	925 (3.9)
None of the above	99 (0.4)	97 (0.4)
Patient declined	31 (0.1)	25 (0.1)

^a^ Cohorts are comprised of all patients carrying a chart diagnosis of a gastrointestinal cancer in the study period (weeks 1–20 of 2019 or 2020). They do not exclusively reflect new diagnoses during this time period. There was no statistically significant difference between cohorts on the basis of sex (χ^2^ = 0.15, *p* = .93) nor race (χ^2^ = 2.82, *p* = .99).

**FIGURE 1:**
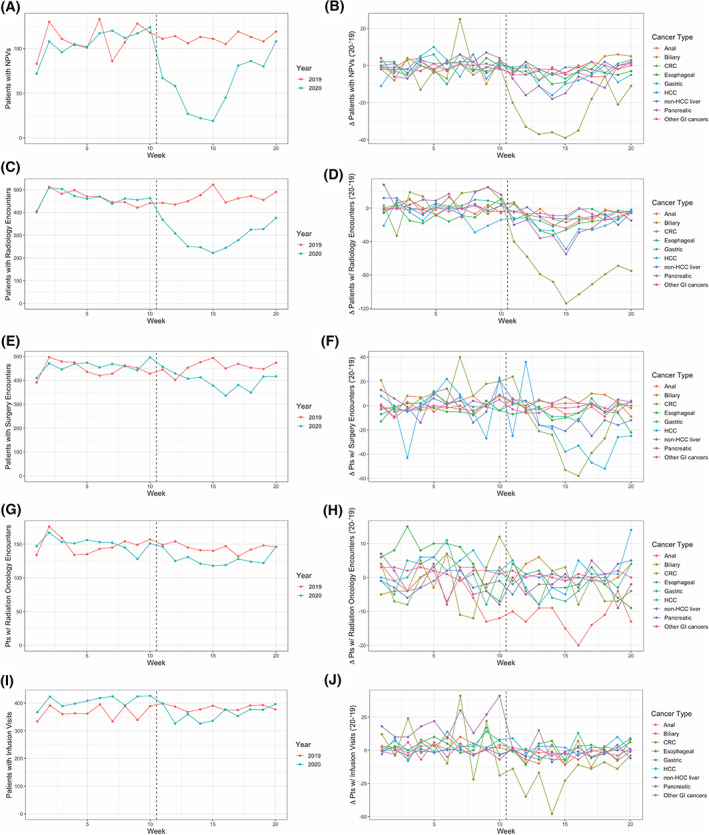
Observed changes in patient encounters among patients with diagnosed GI cancer during the early pandemic period. Column 1 (A, C, E, G, I) includes graphical representations of the number of patients with the corresponding encounter subtype in a given week. Data from 2019 and 2020 are co‐plotted according to the legend appearing at the right of each subpanel. Column 2 (B, D, F, H, J) includes graphical representations of the difference in the number of patients with a recorded encounter subtype within a given week as compared between 2019 and 2020. Data from individual cancer subtypes are co‐plotted according to the legend appearing at the right of each subpanel. The dashed vertical line in both column 1 and 2 corresponds to the beginning of the pandemic period in 2020

When analyzed collectively, the early pandemic period was associated with significant changes in care delivery for patients with a GI malignancy. A total of 49.8 fewer patients had NPVs in a given week (44.9% of the 2019 average) during the early pandemic period than would have otherwise been expected, representing a statistically significant decrease that could not be explained by week‐specific or year‐specific effects alone (*p* = 1.2 × 10^−4^; Figure 1, Table 2). This was seen across all parts of the health system, and among patients with a GI malignancy, the early pandemic period was associated with otherwise unexplained decreases in Radiology encounters (−175.3 patients/week, 38.0% of the 2019 average), Surgery encounters (−70.3 patients/week, 15.6% of the 2019 average), Radiation Oncology encounters (−18.1 patients/week, 12.4% of the 2019 average), and infusion visits (−62.0 patients/week, 16.6% of the 2019 average; Figure 1, Table 2). These findings were all significant after correction for week‐specific and year‐specific effects (Radiology: *p* = 1.9 × 10^−7^, Surgery: *p* = 1.6 × 10^−3^, Radiation Oncology: *p* = 4.1 × 10^−3^, infusion visits: *p* = 6.1 × 10^−5^).

**TABLE 2:** Difference in the weekly frequency of patient encounters during the early pandemic period

Cancer type	NPV				Radiology				Surgery			
	'19 avg^a^	Modeled deficit (pts/wk)^b^	Modeled deficit (% of '19 average)	*p*‐value	'19 avg^a^	Modeled deficit (pts/wk)^b^	Modeled deficit (% of '19 average)	*p*‐value	'19 avg^a^	Modeled deficit (pts/wk)^b^	Modeled deficit (% of '19 average)	*p*‐value
Total	111	−49.8	−44.9	1.2 × 10^−4^	461.6	−175.3	−38.0	1.9 × 10^−7^	451.6	−70.3	−15.6	1.6 × 10^−3^
Anal	5.30	−2.3	−43.4	.12	19.3	−8.9	−46.1	.013	20.9	−3.3	−15.8	.18
Biliary	6.75	0.7	10.4	.74	33.2	−9.3	−28.1	7.7 × 10^−3^	22.7	3.2	14.1	.15
CRC	48.3	−25.1	−52.0	7.0 × 10^−5^	190.4	−86.4	−45.4	1.0 × 10^−8^	162.1	−31.9	−19.7	2.7 × 10^−3^
Esophageal	10.1	−3.2	−31.7	.069	42.3	−10.4	−24.6	.020	32.8	−4.3	−13.1	.12
Gastric	6.75	−2.9	−43.0	.11	24.1	−11.2	−46.5	9.8 × 10^−4^	20.1	−1.9	−9.5	.48
HCC	15.0	−6.3	−42.0	.029	81.2	−11.0	−13.5	.059	152.5	−24.3	−15.9	.027
non‐HCC liver	3.60	−1.5	−41.7	.21	16.1	−11.6	−72.0	4.0 × 10^−4^	40.4	−12.8	−31.7	7.6 × 10^−3^
Pancreatic	19.1	−10.4	−54.5	7.7 × 10^−4^	74.9	−35.8	−47.8	1.2 × 10^−5^	28.4	−2.0	−7.0	.50
Other GI cancers	2.70	−0.9	−33.3	.32	11.6	−5.7	−49.1	2.4 × 10^−3^	6.9	−0.3	−4.3	.85

Cancer Type	Radiation oncology				Infusion visit			
	'19 avg^a^	Modeled deficit (pts/wk)^b^	Modeled deficit (% of '19 average)	*p*‐value	'19 avg^a^	Modeled deficit (pts/wk)^b^	Modeled deficit (% of '19 average)	*p*‐value
Total	146.5	−18.1	−12.4	4.1 × 10^−3^	374.4	−62.0	−16.6	6.1 × 10^−5^
Anal	23.0	−8.1	−35.2	2.2 × 10^−3^	11.3	−5.4	−47.8	4.1 × 10^−3^
Biliary	9.9	1.0	10.2	.55	31.1	−4.0	−12.9	.17
CRC	47.2	−1.9	−4.0	.54	181.9	−23.5	−12.9	6.4 × 10^−3^
Esophageal	24.0	−7.4	−30.8	8.0 × 10^−3^	28.6	−3.2	−11.2	.29
Gastric	7.7	−2.9	−37.7	.18	24.2	−4.9	−20.3	.10
HCC	20.7	−2.2	−10.6	.33	11.0	0.5	4.5	.82
non‐HCC liver	5.2	2.0	38.8	.10	4.0	−1.7	−43.0	.17
Pancreatic	20.2	0.0	0.0	1.00	95.1	−20.2	−21.2	5.5 × 10^−5^
Other GI cancers	0.5	−1.2	−267	.026	10.0	−1.6	−16.0	.44

^a^ The 2019 averages reflect the average number of patients with encounters per week over the period corresponding to the linear model (weeks 1–20).

^b^ The modeled deficit reflects the otherwise unexplained decrease in the number of patients with encounters per week in a difference‐in‐difference linear model accounting for week‐specific and year‐specific effects.

Subgroup analyses revealed cancer‐specific variation in the effect size of changes to care delivery during the early pandemic period. Comparing the weekly count of patients with NPVs revealed independently significant declines most pronounced among patients diagnosed with CRC (−25.1 patients/week, 52.0% of the 2019 average, *p* = 7.1 × 10^−5^), pancreatic cancer (−10.4 patients/week, 54.5% of the 2019 average, *p* = 7.7 × 10^−4^), or HCC (−6.3 patients/week, 42.0% of the 2019 average, *p* = .029; Figure 1, Table 2). Among patients with anal cancer, biliary cancer, esophageal cancer, gastric cancer, non‐HCC liver cancer, or other GI (C17.*, C26.*) cancers, there was not a statistically significant decrease in new patient visits during the early pandemic period.

The number of patients with Radiology encounters significantly decreased amongst all GI cancer subtypes, with the exception of patients with HCC (*p* = .059). The magnitude of this decrease (relative to respective 2019 averages) was most prominent amongst patients diagnosed with non‐HCC liver cancer (−11.6 patients/week, 72.0% of the 2019 average, *p* = 4.0 × 10^−4^). The significance of this decrease was most prominent amongst patients diagnosed with CRC (−86.4 patients/week, 45.4% of the 2019 average, *p* = 1.0 × 10^−8^).

The number of patients with Surgery encounters significantly decreased amongst patients with CRC (−31.9 patients/week, 19.7% of the 2019 average, *p* = 2.7 × 10^−3^), non‐HCC liver cancer (−12.8 patients/week, 31.7% of the 2019 average, *p* = 7.6 × 10^−3^), and HCC (−24.3 patients/week, 15.9% of the 2019 average, *p* = .027). Among patients with anal cancer, biliary cancer, esophageal cancer, pancreatic cancer, gastric cancer, or other GI (C17.*, C26.*) cancers, there was not a statistically significant decrease in Surgery encounters during the early pandemic period.

The number of patients with Radiation Oncology encounters significantly decreased amongst patients with anal cancer (−8.1 patients/week, 35.2% of the 2019 average, *p* = 2.2 × 10^−3^), esophageal cancer (−7.4 patients/week, 30.8% of the 2019 average, *p* = 8.0 × 10^−3^), or other GI (C17.*, C26.*) cancers (−1.2 patients/week, 267% of the 2019 average, *p* = .026). Among patients with biliary cancer, CRC, gastric cancer, HCC, non‐HCC liver cancer, or pancreatic cancer there was not a statistically significant decrease in Radiation Oncology encounters during the early pandemic period.

The number of patients with infusion visits significantly decreased amongst patients with pancreatic cancer (−20.2 patients/week, 21.2% of the 2019 average, *p* = 5.5 × 10^−5^), anal cancer (−5.4 patients/week, 47.8% of the 2019 average, *p* = 4.1 × 10^−3^), or CRC (−23.5 patients/week, 12.9% of the 2019 average, *p* = 6.4 × 10^−3^; Figure 1, Table 2). Among patients with biliary cancer, esophageal cancer, gastric cancer, HCC, non‐HCC liver cancer, or other GI (C17.*, C26.*) cancers, there was not a statistically significant decrease in infusion visits during the early pandemic period.

**FIGURE 2:**
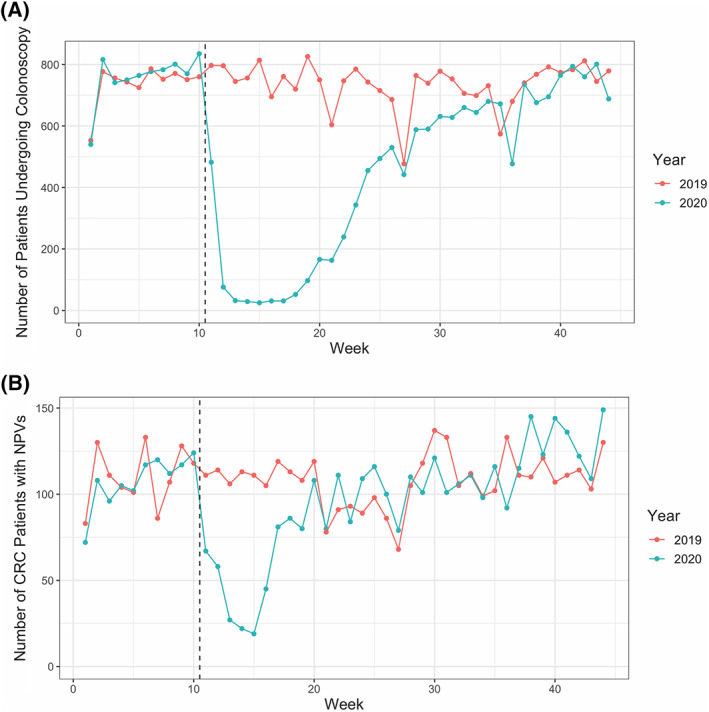
Observed changes in colonoscopy frequency during the early pandemic period. (A) A graphical representation of the number of patients receiving a colonoscopy in a given week in 2019 or 2020. (B) A graphical representation of the number of patients who had a NPV for CRC in a given week in 2019 or 2020. The dashed vertical line in both A and B corresponds to the beginning of the pandemic period in 2020

As highlighted above, patients with CRC had independently significant reductions in NPVs, Radiology encounters, Surgery encounters, and infusion visits. The number of patients undergoing colonoscopies in the health system was queried as a possible enabling etiology for these findings. Significantly fewer patients underwent colonoscopies during the early pandemic period (−684.2 patients/week, 91.0% of the 2019 average, *p* = 5.5 × 10^−12^) (Figure 2, Table 3). By the end of week 20, there were 6436 fewer patients who underwent colonoscopies in 2020 as compared to 2019 (Table 3). By Week 44, this cumulative deficit grew to 9659 patients despite increases in colonoscopy rates (Table 3). This deficit parallels the change seen in NPVs for CRC. Indeed, the number of patients obtaining a colonoscopy was significantly correlated with the number of NPVs in a given week as measured across the first 44 weeks of 2020 (r^2^ = 0.629, *p* = 1.4 × 10^−10^) and the pandemic period alone (weeks 11‐44, r^2^ = 0.722, *p* = 2.1 × 10^−10^) (Figure 2).

**TABLE 3:** Difference in the number of patients who underwent colonoscopies performed during the early pandemic period

	'19 Avg^a^	Modeled deficit (pts/wk)^b^	Modeled deficit (% of '19 average)	*p*‐value	Cumulative ('19)	Cumulative ('20)	Absolute difference
Week 20	751.7	−684.2	−91.0	5.5 × 10^−12^	15 034	8598	−6436
Week 44	736.5	−310.4	−42.1	1.5 × 10^−3^	32 408	22 749	−9659

^a^ The 2019 averages reflect the average number of patients with encounters per week over the period corresponding to the linear model (weeks 1–20).

^b^ The modeled deficit reflects the otherwise unexplained decrease in the number of patients with colonoscopies per week in a difference‐in‐difference linear model accounting for week‐specific and year‐specific effects.

## 4 DISCUSSION

Decreases in overall NPVs, emergency room visits, and hospital admissions during the initial wave of the COVID‐19 pandemic have been reported throughout the United States, highlighting a growing concern that patients have delayed care for conditions that may result in poor outcomes if untreated.^4^, ^5^ From March through May, when the initial state imposed stay at home orders went into effect, the number of NPVs and patient encounters with presumed diagnostic or therapeutic intent (encounters within Radiology, Surgery, and Radiation Oncology departments or classified as an infusion visit) significantly decreased among patients with GI malignancies at an academic medical center, after accounting for temporal trends. Although decreases in healthcare utilization were seen in the majority of GI malignancy types, decreases in all types of medical encounters were not uniform across all GI cancer subtypes, which may have implications for cancer care in the post‐pandemic period. It is likely patients that patients diagnosed with malignancies over the next few months may represent a different patient population than those typically seen by oncologists in the United States prior to the delays in care associated with the pandemic.

While overall decreases in NPVs and encounter types were seen in patients with all GI cancers, subgroup analyses by cancer type showed disease specific variation in treatment modalities and may have potential implications for the resurgence. For example, as pancreatic cancer typically presents with symptomatic or incidentally caught late‐stage disease, pancreatic cancer presentation, and subsequent initial urgent treatment may not be as affected by the disruption of health care services from COVID‐19. Similarly, no significant decreases in surgical department encounters were seen in esophageal and anal cancers, which also tend to present at higher stages and with a greater symptomatic burden. This is corroborated by the relatively low impact seen on surgical and radiation oncology encounters for patients diagnosed with pancreatic cancer. In our study, however, patients with pancreatic cancer were found to have significantly fewer radiology and infusion encounters than expected. This trend is concerning as research suggests that the timing of chemotherapy administration affects pancreatic cancer outcomes.^6^ Contributing factors likely include the temporary freeze of clinical trial enrollment at our large academic center during the pandemic and subsequent patient preference to undergo standard of care chemotherapy options closer to home and in the community.

Perhaps most worrisome is that some of the greatest decreases in NPVs and specialty encounters were seen in CRC, the only gastrointestinal cancer that is effectively and universally screened for, and as a result, can often be detected in earlier stages than other GI cancers. These decreases were observed in the setting of the precipitous decline in patients undergoing colonoscopy, amounting to an unexplained deficit equivalent to 91% of the 2019 average. These results are consistent with recent surveys of gastroenterology departments across the United States and the UK reporting 41.7%–84.5% decreases in CRC screening.^7^, ^8^, ^9^ Delays in CRC screening and surgical resection have been associated with advanced stage of disease on presentation and worse survival outcomes.^10^, ^11^ Unfortunately, yet predictably, these data indicate a strong correlation between the number of colonoscopies across the health system and the number of NPVs in patients ultimately diagnosed with CRC. Though colonoscopies have begun to restart and increase in volume at this and other medical centers, there is still a significant deficit in colonoscopies compared to the prior year (9659 as of week 44), and the effects of backlogged cancer screening appointments may still lead to diagnostic delays that may lead to worse long‐term outcomes, including upstaging of patients. In addition, we found widespread decreases in radiology encounters across all but one cancer type. Studies from the UK and the Netherlands show reductions in both the number of endoscopies and GI cancers detected ranging from 50% to 88%.^12^, ^13^ Altogether, these results suggest that all forms of routine surveillance scans and monitoring are also backlogged and may contribute to further diagnostic or therapeutic delays. As seen in Sozutek et al, a prospective study of 177 GI cancer patients who underwent elective surgery without delay from March ‐ November 2020, the risk of delaying procedures may be significantly greater than the risk of contracting COVID‐19 with proper isolation measures.^14^

One limitation of our study is that the decreases seen in a single academic institution may not be generalizable to other sites. However, it is likely that due to the highly multidisciplinary nature of gastrointestinal cancer care, that these trends parallel those observed in similar academic health systems. In addition, our study's reliance upon our electronic health record's reporting tools, which were used to generate aggregate weekly metrics, precluded data reporting on an individual patient‐level basis. Ongoing and future work at our institution will examine more detailed, patient‐level trends, and trends in cancer staging will be interesting to examine as we move beyond the current pandemic.

It remains to be seen what the ultimate impact on patient survival and outcomes will be due to the COVID‐19 pandemic, as many pertinent endpoints will take time to mature. It will also take time to fully determine the impact of delays in screening will have on factors such as patients' stage at presentation. Nonetheless, this study demonstrates a significant reduction in health encounters pertinent to the detection, diagnosis, treatment, and management of GI malignancy. Most striking is the significant impact on CRC screening, potentially adversely affecting outcomes for a disease that is treatable and curable, especially in the early stages. Although it may be too early to ascertain the full impact of COVID on gastrointestinal cancer care and outcomes, one clear area of focus may be to increase the availability and access to colonoscopy screenings as hospital systems continue to adapt to the COVID‐19 pandemic and expand health system capacities.

## CONFLICT OF INTEREST

The authors have stated explicitly that there are no conflicts of interest in connection with this article.

## ETHICAL STATEMENT

This study was approved by the “Blinded for Peer Review” Institutional. Review Board with a waiver of the HIPAA authorization requirement.

## AUTHORS' CONTRIBUTIONS

All authors had full access to the data in the study and take responsibility for the integrity of the data and the accuracy of the data analysis. *Conceptualization*, N.R.P., C.K., E.B.‐J., J.P.P., L.N.S., A.W.; *Data Curation*, N.R.P., C.K., C.B., L.J.K., P.G.; *Methodology*, N.R.P., C.K., L.J.K., C.S., U.T., P.G., A.W.; *Project Administration*, C.S., U.T., L.N.S., A.W.; *Investigation*, N.R.P., C.K.; *Formal Analysis*, N.R.P., C.K.; *Writing ‐ Original Draft*, N.R.P., C.K.; *Writing ‐ Review & Editing*, N.R.P., C.K., L.J.K., C.S., U.T., E.B.‐J., P.G., J.P.P., L.N.S., A.W.; *Visualization*, N.R.P., C.K.; *Supervision*, A.W.

## Data Availability

The data that support the findings of this study are available from the corresponding author upon reasonable request.
